# Long noncoding RNA HULC contributes to paclitaxel resistance in ovarian cancer via miR-137/ITGB8 axis

**DOI:** 10.1515/biol-2021-0058

**Published:** 2021-07-01

**Authors:** Bo Huang, Min Wei, Li Hong

**Affiliations:** Department of Gynaecology and Obstetrics, Hubei General Hospital, No. 99 Zhang-Zhi-Dong Street, Wuchang District, Wuhan 430000, Hubei, China

**Keywords:** lncRNA HULC, miR-137, ITGB8, paclitaxel resistance, ovarian cancer

## Abstract

Long noncoding RNA (lncRNA) highly upregulated in liver cancer (HULC) has been reported to be implicated in chemoresistance. However, the potential mechanism of HULC in paclitaxel (PTX)-resistant ovarian cancer (OC) remains undefined. The expression of RNAs and proteins was measured by quantitative reverse transcriptase polymerase chain reaction (qRT-PCR) and Western blot assay. The PTX resistance and apoptotic rate were assessed via 3-(4,5-dimethylthiazol-2-yl)-2,5-diphenyltetrazolium bromide (MTT) assay and flow cytometry, respectively. Furthermore, the interaction between miR-137 and HULC or integrin beta-8 (ITGB8) was predicted by miRcode and starBase v2.0 and then verified by dual luciferase reporter and RNA pull-down assays. In addition, the xenograft mice model was established to explore the effects of HULC *in vivo*. HULC was significantly upregulated and miR-137 was downregulated in PTX-resistant OC tissues and cells. Also, the HULC depletion suppressed tumor growth and PTX resistance in PTX-treated mice. miR-137 was verified as a target of HULC and directly targeted ITGB8. And HULC knockdown downregulated ITGB8 expression by targeting miR-137. miR-137 inhibitor or ITGB8 overexpression mitigated the suppressive impacts of HULC knockdown on PTX resistance. Collectively, HULC modulated ITGB8 expression to promote PTX resistance of OC by sponging miR-137.

## 1 Introduction

Ovarian cancer (OC) is one of the most lethal gynecologic malignancies with poor prognosis. Due to the insidious onset and indistinct symptoms as well as the lack of effective screening approaches, 70% of patients are often diagnosed in the late stages [1,2]. Nowadays, surgery, chemotherapy, and radiotherapy are three main approaches for OC therapy. Paclitaxel (PTX), an antineoplastic agent, which inhibits cellular mitosis and plays a vital role in the therapy of cancer, is recommended as a typical first-line treatment for OC [3]. However, most patients were subjected to relapse after surgery or develop resistance to chemotherapy drugs [4]. Thus, PTX contributes greatly to the OC treatment and often determines the prognosis of patients. The aim of this study is to investigate the mechanism of PTX resistance in OC.

Long noncoding RNA (lncRNA), a type of RNA (>200 nucleotides [nts]) with no potential to encode proteins or short peptides, was reported to function as a competing endogenous RNA (ceRNA) to competitively bind to microRNAs (miRNAs), thereby regulating gene expression at the transcription and posttranscriptional levels [5,6]. Previous research has disclosed that dysregulation of lncRNAs was associated with chemotherapy resistance in OC [7,8]. For example, lncRNA small nucleolar RNA host gene 22 (SNHG22) overexpression enhanced the chemotherapy resistance of epithelial ovarian carcinoma via interacting with miR-2467/Gal-1 axis, and PI3K/AKT and ERK pathway [9]. Wang et al. indicated that colon cancer-associated transcript 1 (CCAT1) acted as a miRNA sponge for miR-454 to regulate Survivin expression, thereby enhancing the cisplatin resistance of OC cells [10]. Besides, downregulation of CHRF reversed the activation of epithelial-to-mesenchymal-transition (EMT) and STAT3 signaling and then improved the sensitivity of OC cells to cisplatin [11]. lncRNA highly upregulated in liver cancer (HULC), located in chromosome 6p24.3, was reported to be aberrantly elevated in several tumors, including human pancreatic cancer [12], gastric cancer [13], osteosarcoma [14], and OC [15]. HULC was considered as a potential biomarker for cancer diagnosis and treatment [12,16]. More importantly, several research studies have uncovered the pivotal role of HULC in chemotherapy resistance in cancers. For example, Xiong et al. indicated that HULC positively regulated ubiquitin-specific peptidase 22 (USP22) expression to stabilize silent information regulator 1 (Sirt1) protein and induce protective autophagy, thereby inhibiting the sensitivity of hepatocellular carcinoma cells to oxaliplatin, 5-fluorouracil, and pirarubicin (THP) treatment [17]. Besides, HULC regulated the miR-150-5p/MCL1 axis to enhance imatinib resistance in chronic myeloid leukemia [18]. However, the function and mechanism underlying HULC in the resistance of chemotherapy of OC are still not well understood.

microRNA is a class of RNA containing ∼22 nts in length with no ability to translate, which were reported to be related to chemoresistance in OC. For instance, Park et al. proved that miR-503-3p was suppressed by CD97 in PTX-resistant OC cells and then promoted the migratory and invasive OC cells [19]. Another study exhibited that OC chemoresistance was sensitized by miR-200c *in vitro* [20]. microRNA-137 (miR-137) is located on human chromosome 1p22 and functions as a tumor suppressor in various types of cancer [21,22,23]. Previous evidence revealed that miR-137 was suppressed by c-Myc and then elevated the expression of EZH2, thus inhibited the sensitivity of OC cells to cisplatin [24]. But the mechanism of miR-137 in PTX resistance of OC remains unclear.

In the present research, the functions and regulatory mechanism underlying HULC and miR-137 in PTX resistance in OC were investigated. We aimed to uncover the mechanism of how HULC and miR-137 modulate PTX resistance in OC, hoping to seek a novel target for PTX-resistant OC treatment.

## 2 Materials and methods

### 2.1 Tissue specimens

A total of 62 OC tissues and adjacent normal tissues were obtained from OC patients who received oophorectomies at Hubei General Hospital. OC patients who were confirmed by the postoperative pathologic examination and only received PTX-based chemotherapy before surgery were enrolled in this study. The clinicopathological parameters of OC patients are shown in Table 1. According to the National Comprehensive Cancer Network guidelines, patients with persistent or recurrent disease within 6 months after completing primary chemotherapy were regarded as PTX-resistant cases (*n* = 24), otherwise, it can be defined as PTX-responsive cases (*n* = 38). All tissues were frozen at −80°C until further usage.

**Table 1:** Correlation between HULC expression and clinicopathological parameters of ovarian patients

Parameter	Case	HULC expression		*P* value
		Low (*n* = 30)	High (*n* = 32)	
**Age (years)**				0.459
≤50	28	15	13	
>50	34	15	19	
**Ascites**				0.469
<100	24	13	11	
≥100	38	17	21	
**Tumor size (cm)**				0.0001*
≤3	26	20	6	
>3	36	10	26	
**TNM stages**				0.002*
I–II	25	18	7	
III	37	12	25	
**Lymphatic metastasis**				0.02*
Negative	32	20	12	
Positive	30	10	20	

TNM, tumor-node-metastasis; *P < 0.05.


**Informed consent:** Informed consent has been obtained from all individuals included in this study.
**Ethical approval:** The research related to human use has been complied with all the relevant national regulations, institutional policies, and in accordance with the tenets of the Helsinki Declaration, and has been approved by the Ethics Committee of Hubei General Hospital.

### 2.2 Cell culture

Human OC cell lines (SKOV3 and HeyA-8) were bought from Huiying (Shanghai, China). The PTX-resistant SKOV3 and HeyA-8 cells were established by stepwise PTX concentrations increasing from 5 to 300 nM over a 6-month period, as previously described [25], and named SKOV3/PTX and HeyA-8/PTX. All cells were cultivated in RPMI-1640 medium (BIOSUN, Shanghai, China) supplemented with 10% fetal bovine serum (Genetimes, Shanghai, China) in 5% CO_2_ incubator at 37°C. In addition, 5 nM PTX was added into the RPMI-1640 medium to maintain the PTX-resistant phenotype of SKOV3/PTX and HeyA-8/PTX cells.

### 2.3 Transient transfection

Small interfering RNA (siRNA) against HULC (si-HULC) and corresponding negative control (si-NC), miR-137 mimics (miR-137) and its negative control (miR-NC), miR-137 inhibitor (anti-miR-137) and its negative control (anti-miR-NC), as well as siRNA targeting ITGB8 (si-ITGB8) were bought from GenePharma (Shanghai, China). SKOV3/PTX and HeyA-8/PTX cells (5 × 10^4^ cells per well) were plated into the six-well plates overnight prior to transfection. Then, the oligonucleotides were transfected into the cells using Lipofectamine^TM^ 2000 (Invitrogen, Carlsbad, CA, USA) according to the manufacturer’s instructions. Following a 48 h transfection, cells were collected for further investigation.

### 2.4 Quantitative reverse transcriptase polymerase chain reaction (qRT-PCR)

Total RNA from OC tissues and cells were extracted using TRIzol reagent (Invitrogen) and then random primers (Invitrogen) were used for reverse transcription. The quantitative PCR was performed using miScript SYBR Green PCR Kit (Qiagen NV, Venlo, Netherlands) on a 96-well Real-Time PCR System (Applied Biosystems, Foster City, USA). The relative expression of HULC and ITGB8 were normalized by internal reference glyceraldehyde 3-phosphate dehydrogenase (GAPDH) and calculated by the 2^−ΔΔCt^ method. And U6 snRNA was utilized for the normalization of miR-137. The primers were obtained from Beijing Genomics Institute (BGI, Shenzhen, China) and listed in Table 2.

**Table 2:** Primer sequences used for qRT-PCR in this research

Gene	Sequences
HULC-F	5′-ACTCTGAAGTAAAGGCCGGA-3′
HULC-R	5′-TGCCAGGAAACTTCTTGCTTG-3′
miR-137-F	5′-GCGCGCTTATTGCTTAAGAATAC-3′
miR-137-R	5′-GCTGTCAACGATACGCTACGTA-3′
ITGB8-F	5′-TGAGGCGAAAAGGACAAGGG-3′
ITGB8-R	5′-TCTGGGAGCGCCCTAGAAG-3′
GAPDH-F	5′-TGTTCGTCATGGGTGTGAAC-3′
GAPDH-R	5′-ATGGCATGGACTGTGGTCAT-3′
U6-F	5′-CTCGCTTCGGCAGCACA-3′
U6-R	5′-AACGCTTCACGAATTTGCGT-3′

Abbreviations: HULC: highly upregulated in liver cancer; ITGB8: integrin beta-8; F: forward; R: reverse.

### 2.5 MTT assay

3-(4,5-Dimethylthiazol-2-yl)-2,5-diphenyltetrazolium bromide (MTT; Sigma-Aldrich, Louis, MO, USA) was used to assess PTX resistance. OC cells (5 × 10^4^ cells per well) were cultured in a 96-well plate for 24 h and then exposed to different concentrations of PTX for another 48 h. Subsequently, 20 μL of MTT (5 mg/mL) was added into each well and incubated for 4 h at 37°C. Then 150 μL of dimethyl sulfoxide (Sigma-Aldrich) was added to each well to dissolve the formazan crystals. Spectrophotometer (Thermo Fisher Scientific, Rockville, MD, USA) was utilized to examine the absorbance at 490 nm. The relative survival curve was drawn to get the 50% inhibition of growth (IC_50_).

### 2.6 Western blot assay

Protein samples (30 μg) from PTX-resistant cells were lysed in RIPA lysis buffer (Beyotime Biotechnology, Shanghai, China) and quantified using the bicinchoninic acid protein assay kit (Beyotime Biotechnology). Then, equal amount of protein samples (30 μg) was separated by 10% sodium dodecyl sulfonate-polyacrylamide gel electrophoresis, and transferred onto polyvinylidene fluoride membranes. Subsequently, the membranes were blocked with 5% (w/v) skim milk for 2 h and then incubated overnight at 4°C with primary antibodies against P-glycoprotein (P-gp, ab235954, 1:1,000), glutathione S-transferase π (GST-π, ab153949, 1:1,500), GAPDH (ab9485, 1:2,000), or integrin beta 8 (ITGB8, ab172007, 1:1,000). Subsequently, the membranes were incubated with horseradish peroxidase-conjugated goat anti-rabbit (ab205718, 1:20,000) or goat anti-mouse (ab205719, 1:15,000) secondary antibodies for 2 h at room temperature. The antibodies were brought from Abcam (Cambridge, MA, USA). The bands were visualized using a chemiluminescent substrate (ECL kit; Beyotime, Shanghai, China). GAPDH was used as the internal normalization control.

### 2.7 Flow cytometry

The apoptosis rate was assessed using Annexin V-fluorescein isothiocyanate (FITC)/propidium iodide (PI) apoptosis detection kit (Solarbio, Beijing, China). Briefly, SKOV3/PTX and HeyA-8/PTX cells (5 × 10^5^ cells) were cultivated in six-well plates at 37°C for 48 h. Then 1 × 10^6^ cells were resuspended in the ice-cold phosphate buffer solution (PBS) and incubated with 5 μL of the Annexin V-FITC and PI solution for 15 min in the dark. The apoptotic rate was monitored using a flow cytometry (Mindray, Shenzhen, China).

### 2.8 Dual luciferase reporter assay

Bioinformatics software starBase v2.0 and miRcode were used to predict the downstream targets of HULC and miR-137. The fragments of HULC and ITGB8 3′-untranslated regions (3′-UTR) harboring the wild type (Wt) or mutant type (Mut) miR-137 binding sites were amplified and inserted into psiCHECK2 vector (Promega, Madison, WI, USA) to contrast the luciferase reporter, namely HULC-Wt, HULC-Mut, ITGB8-Wt, or ITGB8-Mut. SKOV3/PTX and HeyA-8/PTX cells were co-transfected with the recombinant reporter vectors and miR-137 or miR-NC using Lipofectamine 2000 (Invitrogen) according to the manufacturer’s guidance. After transfection for 48 h, the luciferase activities were detected using a luciferase activity detect kit (Promega).

### 2.9 RNA pull-down assay

RNA pull-down assay was conducted using the Pierce Magnetic RNA-Protein Pull-Down kit (Thermo Fisher Scientific, Waltham, MA, USA) following the manufacturer’s instructions. Biotin-labelled miR-137 (Bio-miR-137), Biotin-labelled mutant miR-137 (mutation at the HULC binding sites, Bio-miR-137-Mut), Biotin-labelled negative control (Bio-miR-NC), Biotin-labelled HULC (Bio-HULC), Biotin-labelled mutant HULC (mutation at the miR-137 binding sites, Bio-HULC-Mut), and Biotin-labelled negative control (Bio-NC) were purchased from Sangon (Shanghai). In brief, SKOV3/PTX and HeyA-8/PTX cells lysate samples were incubated with Bio-miR-137, Bio-miR-137-Mut, Bio-miR-NC, Bio-HULC, Bio-HULC-Mut, or Bio-NC. Then, the RNA–RNA complex was conjugated with streptavidin magnetic beads. After elution, the enrichments of HULC and miR-137 in SKOV3/PTX and HeyA-8/PTX cells were measured by qRT-PCR.

### 2.10 Mice xenograft models

Recombinant lentivirus carrying short hairpin RNA (shRNA) against HULC (sh-HULC) or negative control (Scrambled) was purchased from Genechem (Shanghai, China). SKOV3/PTX cells were infected with sh-HULC or Scrambled lentivirus and then subjected to selection in 4 μg/mL puromycin for several weeks. Six-week-old nude mice (*n* = 6 per group) were obtained from Shanghai Laboratory Animal Center (Shanghai, China). Stable SKOV3/PTX cells (3 × 10^6^/100 µL PBS) expressing sh-HULC or Scrambled were subcutaneously injected into the right side of the nude mice. After 7 days of inoculation, mice were intraperitoneally injected with PTX (3 mg/kg) or PBS every 3 days for 28 days. The mice were divided into four groups, namely, group 1: Scrambled, group 2: sh-HULC, group 3: PTX, and group 4: sh-HULC + PTX. The tumor volume was measured every 3 days for 28 days, and calculated with the formula: volume (mm^3^) = width^2^ × length/2. After 28 days, all mice were sacrificed, and tumor tissues were harvested and weighed. The excised xenograft tumor samples were snap frozen at −80°C for further qRT-PCR analysis.


**Ethical approval:** The research related to animal use has been complied with all the relevant national regulations and institutional policies for the care and use of animals, and has been approved by the Animal Care Committee of Hubei General Hospital.

### 2.11 Statistical analysis

The data were analyzed by GraphPad Prism 7 (GraphPad Inc., La Jolla, CA, USA) and displayed as mean ± standard deviation. Student’s *t*-test and one-way analysis of variance were used to analyze the comparisons between two groups or among multiple groups. *P* value less than 0.05 was considered as a significant difference.

## 3 Results

### 3.1 HULC expression was enhanced while miR-137 expression was decreased in PTX-resistant OC tissues and cells

**Figure 1:**
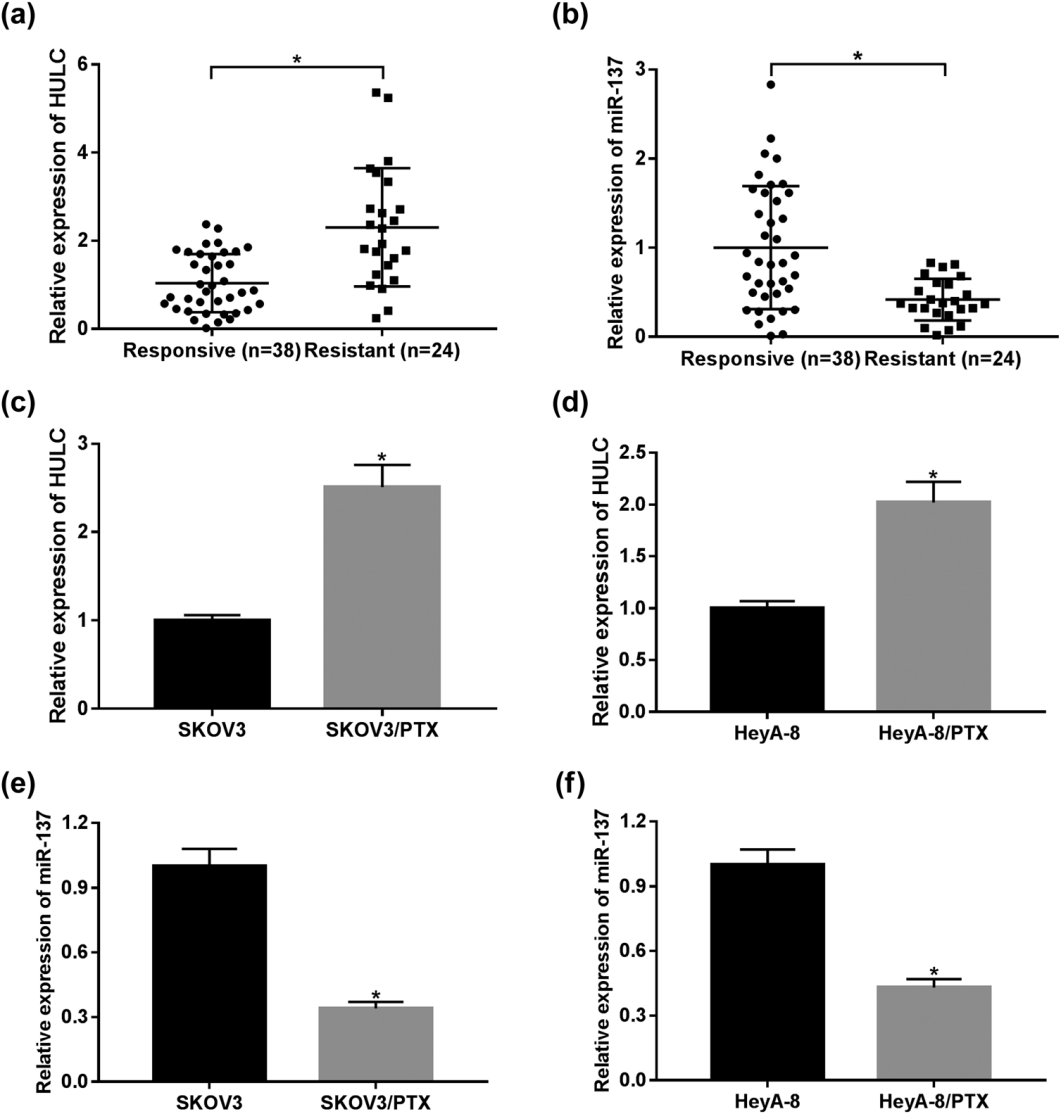
HULC expression was enhanced while miR-137 expression was decreased in PTX-resistant OC tissues and cells. (a and b) The levels of HULC (a) and miR-137 (b) in PTX-responsive (*n* = 38) and PTX-resistant (*n* = 24) OC tissues were detected via qRT-PCR. (c and d) The expression level of HULC in PTX-resistant SKOV3 (c) and HeyA-8 (d) cells as well as PTX-responsive SKOV3 and HeyA-8 cells was measured by qRT-PCR. (e and f) The expression of miR-137 in PTX-resistant SKOV3 (e) and HeyA-8 (f) cells as well as PTX-responsive SKOV3 and HeyA-8 cells was assessed by qRT-PCR. *P < 0.05.

**Figure A1:**
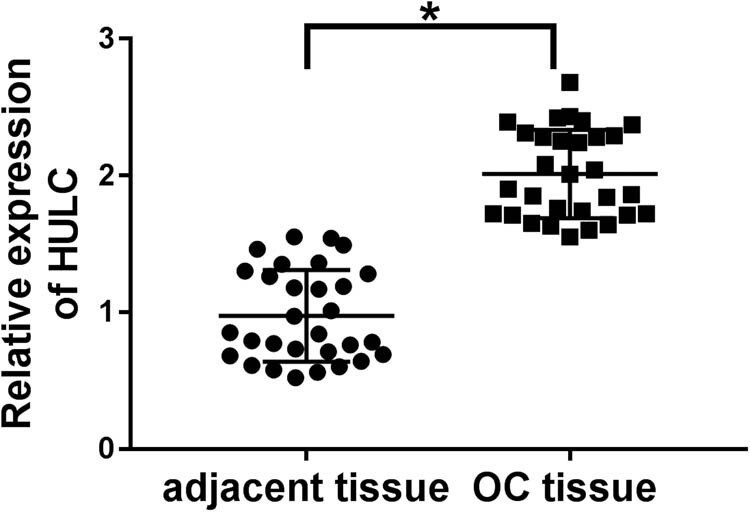
HULC was highly expressed in OC tissues. The expression levels of HULC in OC tissues and adjacent normal tissues were detected by qRT-PCR. *P < 0.05.

As mentioned in Section 1, HULC was highly expressed in OC tissues compared with the adjacent normal tissues (Figure A1), and its high expression was positively related to tumor size (*P* = 0.0001), TNM stages (*P* = 0.002), and lymphatic metastasis (*P* = 0.02) in patients with OC (Table 1). However, its functional effect in PTX-resistant OC remains unclear. To explore the expression levels of HULC and miR-137 in PTX-resistant OC, qRT-PCR analysis was performed in PTX-responsive (*n* = 38) and PTX-resistant (*n* = 24) OC tissues. As shown in Figure 1a and b, the expression level of HULC was significantly upregulated, while miR-137 was distinctly downregulated in PTX-resistant OC tissues in comparison with that in PTX-sensitive OC tissues. Similarly, HULC expression level was also increased (Figure 1c and d), and miR-137 was decreased (Figure 1e and f) in PTX-resistant OC cells (SKOV3/PTX and HeyA-8/PTX) compared to that in PTX-sensitive OC cells (SKOV3 and HeyA-8). These data revealed the aberrant expression of HULC and miR-137 in PTX-resistant OC tumors and cells.

### 3.2 HULC knockdown enhanced the PTX sensitivity of PTX-resistant OC cells *in vitro*

**Figure 2:**
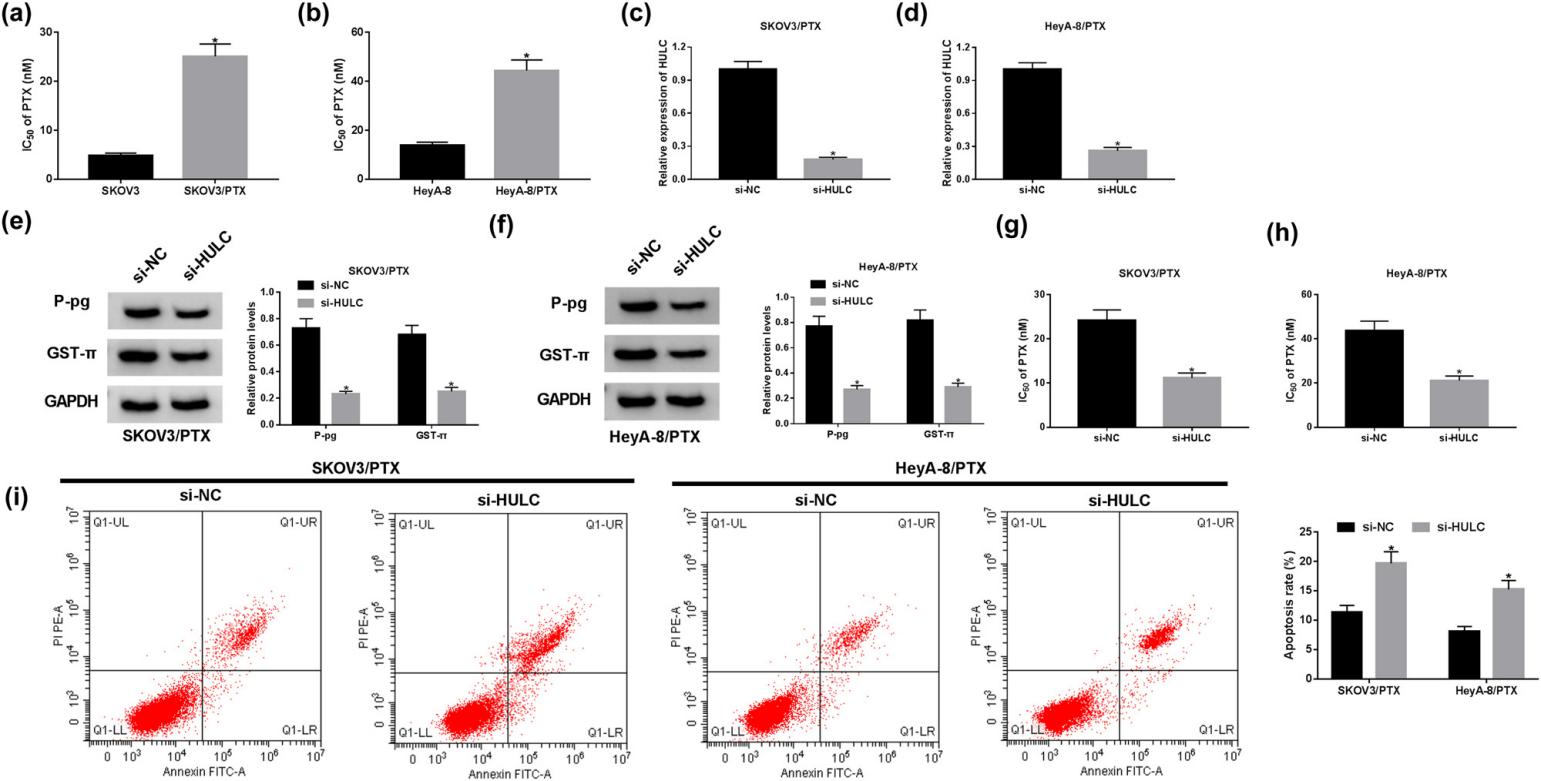
HULC knockdown enhanced the PTX sensitivity of PTX-resistant OC cells *in vitro*. (a and b) IC_50_ values of SKOV3/PTX or SKOV3 cells (a) as well as HeyA-8/PTX and HeyA-8 cells (b) against PTX were calculated by MTT assay. (c–i) The SKOV3/PTX and HeyA-8/PTX cells were transfected with si-HULC or si-NC, respectively. (c and d) The expression level of HULC in transfected SKOV3/PTX (c) and HeyA-8/PTX (d) cells was measured by qRT-PCR. (e and f) The protein levels of P-gp and GST-π in transfected SKOV3/PTX (e) and HeyA-8/PTX (f) cells were detected by western blot. (g and h) IC_50_ values of transfected SKOV3/PTX (g) and HeyA-8/PTX (h) cells against PTX were assessed by MTT assay. (i) The apoptosis rate of transfected SKOV3/PTX and HeyA-8/PTX cells was evaluated by flow cytometry assay. *P < 0.05.

Compared to the PTX-sensitive OC cells (SKOV3 and HeyA-8), the IC_50_ values for PTX-resistant OC cells increased three- to five-fold (Figure 2a and b), which indicated the successful establishment of PTX-resistant OC cells. To explore the function of HULC in OC, si-HULC was transfected into PTX resistance to alter HULC expression. As shown in Figure 2c and d, the expression level of HULC declined more than 70% in PTX-resistant OC cells transfected with si-HULC. Several drug transporter proteins in tumor cells, including P-gp and GST-π, are involved in the chemotherapy resistance [26,27]. We further investigated the protein levels of P-gp and GST-π in PTX-resistant OC cells with HULC knockdown. The protein levels of P-gp and GST-π were obviously decreased in the si-HULC group in contrast with that in the si-NC group (Figure 2e and f). Furthermore, transfection of si-HULC markedly reduced the IC_50_ values of SKOV3/PTX and HeyA-8/PTX cells against PTX (Figure 2g and h). Besides, flow cytometry assay presented that the apoptosis rate was effectively upregulated in SKOV3/PTX and HeyA-8/PTX cells transfected with si-HULC (Figure 2i). Taken together, these results demonstrated that depletion of HULC enhanced the sensitivity of PTX-resistant OC cells to PTX *in vitro*.

### 3.3 miR-137 was a direct target of HULC

**Figure 3:**
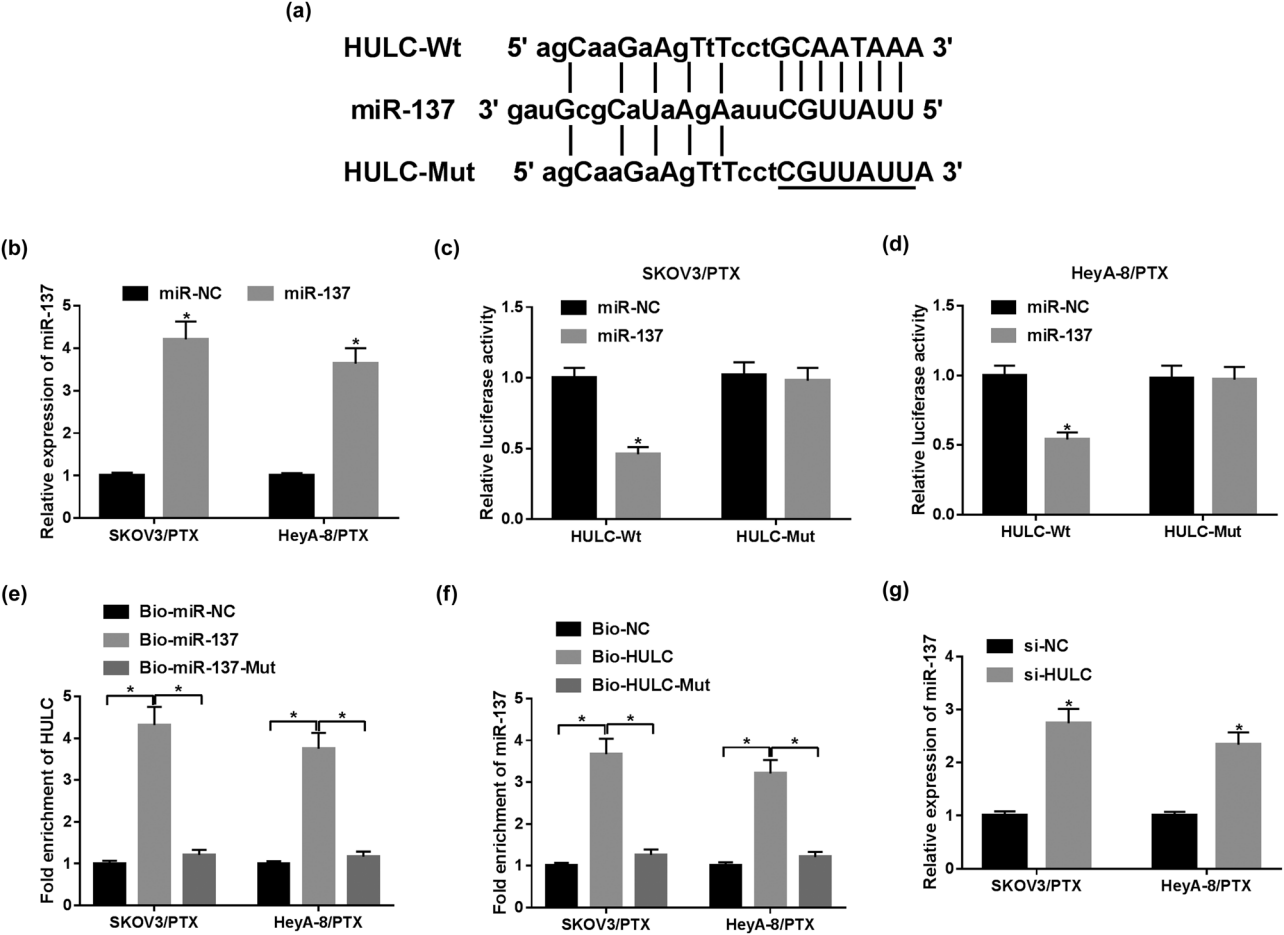
miR-137 was a direct target of HULC. (a) The complementary binding sites between miR-137 and HULC are shown. (b) The expression level of miR-137 in SKOV3/PTX and HeyA-8/PTX cells transfected with miR-137 or miR-NC was detected by qRT-PCR assay. (c and d) The luciferase activity of SKOV3/PTX (c) and HeyA-8/PTX (d) cells co-transfected with HULC-Wt or HULC-Mut vectors and miR-NC or miR-137. (e) RNA pull-down assay was employed to assess the enrichment of HULC in SKOV3/PTX and HeyA-8/PTX cells treated with Bio-miR-NC, Bio-miR-137, or Bio-miR-137-Mut. (f) The enrichment of miR-137 in SKOV3/PTX and HeyA-8/PTX cells treated with Bio-NC, Bio-HULC, or Bio-HULC-Mut was monitored by RNA pull-down assay. (g) The expression level of miR-137 in SKOV3/PTX and HeyA-8/PTX cells transfected with si-HULC or si-NC was measured by qRT-PCR. *P < 0.05.

To investigate the biological mechanism of HULC in PTX-resistant OC cells, miRcode online database (http://mircode.org) was utilized to search for the putative target of HULC. The results showed that the HULC sequence contained the complementary binding sites of miR-137 (Figure 3a). To verify the target relationship between HULC and miR-137, dual luciferase reporter and RNA pull-down assays were conducted. The expression level of miR-137 in PTX-resistant OC cells was elevated by four times in cells with miR-137 mimic transfection compared with the miR-NC group (Figure 3b). Dual luciferase reporter assay demonstrated that transfection of miR-137 decreased the luciferase activity of the HULC-Wt group in contrast with the miR-NC group, while the luciferase activity of the HULC-Mut group had no apparent change (Figure 3c and d). Furthermore, the RNA pull-down assay presented that HULC was substantially enriched by Bio-miR-137 in SKOV3/PTX and HeyA-8/PTX cells compared with that in the Bio-miR-NC group (Figure 3e). Similarly, miR-137 was significantly enriched by Bio-HULC (Figure 3f). Furthermore, the enrichment of miR-137 and HULC was greatly reduced in cells incubated with the mutant miR-137 probe (Bio-miR-137-Mut) or HULC probe (Bio-HULC-Mut) (Figure 3e and f), which confirmed the direct interaction between HULC and miR-137 in PTX-resistant OC cells. Besides, the miR-137 level in SKOV3/PTX and HeyA-8/PTX cells was significantly increased by transfection of si-HULC (Figure 3g). Altogether, HULC negatively regulated miR-137 by sponging miR-137 in PTX-resistant OC cells.

### 3.4 HULC depletion enhanced PTX sensitivity in PTX-resistant OC cells by negatively regulating miR-137

**Figure 4:**
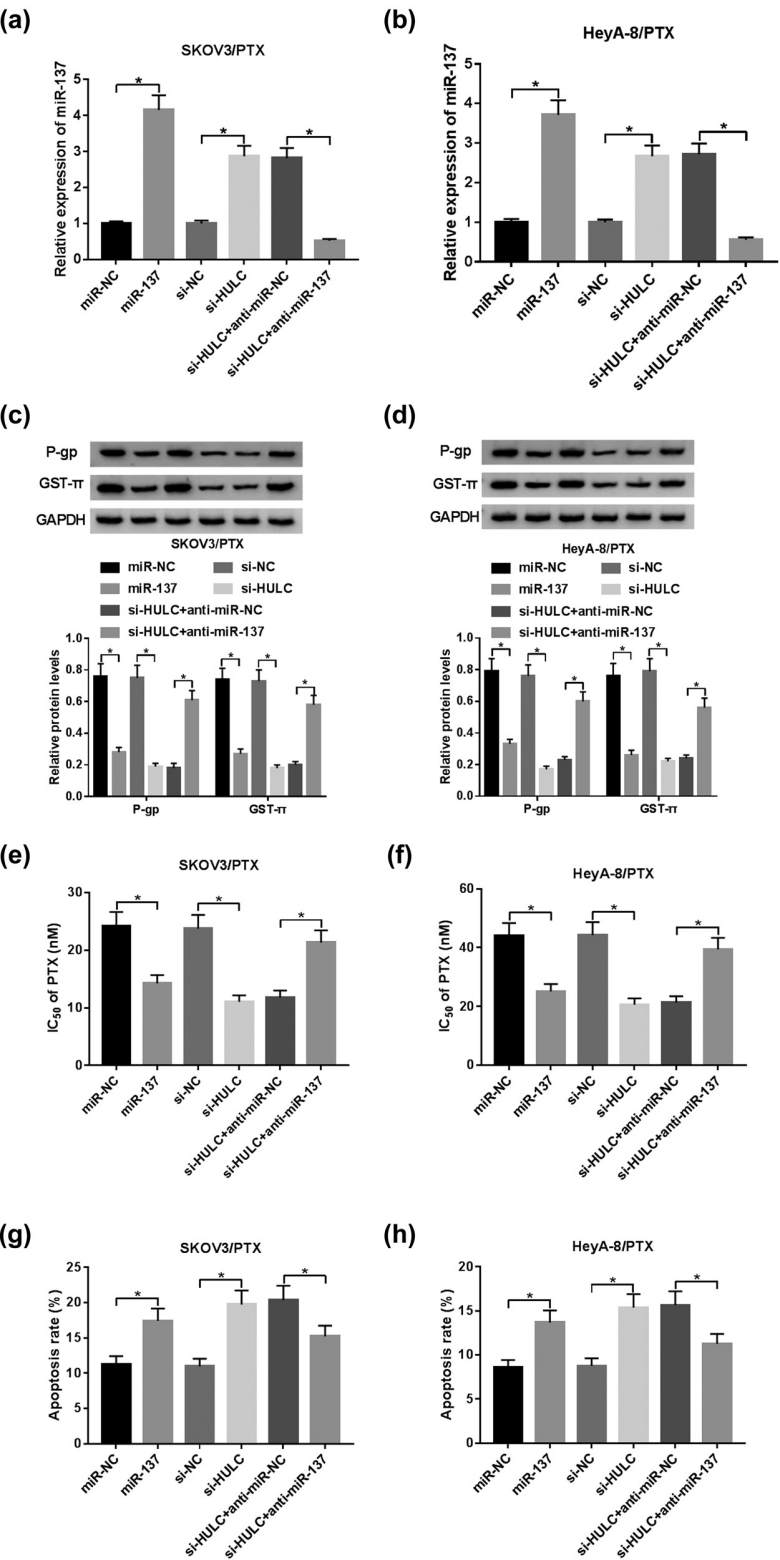
HULC depletion enhanced PTX sensitivity by negatively regulating miR-137 in PTX-resistant OC cells. The SKOV3/PTX and HeyA-8/PTX cells were transfected with miR-NC, miR-137, si-NC, si-HULC, si-HULC + anti-miR-NC, or si-HULC + anti-miR-137, respectively. (a and b) The expression level of miR-137 in transfected SKOV3/PTX (a) and HeyA-8/PTX (b) cells was measured by qRT-PCR. (c and d) The protein levels of P-gp and GST-π in transfected SKOV3/PTX (c) and HeyA-8/PTX (d) cells were detected by western blot. (e and f) IC_50_ values of transfected SKOV3/PTX (e) and HeyA-8/PTX (f) cells against PTX were calculated by MTT assay. (g and h) The apoptosis rate of transfected SKOV3/PTX (g) and HeyA-8/PTX (h) cells was evaluated by flow cytometry assay. *P < 0.05.

To examine whether miR-137 was involved in the regulation of HULC on PTX resistance, loss-of-function assays were performed in SKOV3/PTX and HeyA-8/PTX cells. As displayed in Figure 4a and b, the miR-137 level was upregulated by miR-137 mimic or HULC knockdown, whereas this tendency was recovered by co-transfection of si-HULC and anti-miR-137. Besides, western blot assay implicated that anti-miR-137 partly reversed the decreasing protein levels of P-gp and GST-π in SKOV3/PTX and HeyA-8/PTX cells mediated by si-HULC or miR-137 mimic (Figure 4c and d). Furthermore, downregulation of miR-137 also reversed the promotion effects of si-HULC or miR-137 mimic on the IC_50_ values of SKOV3/PTX and HeyA-8/PTX against PTX (Figure 4e and f). In addition, miR-137 inhibitor attenuated the facilitated impacts on apoptotic rate in SKOV3/PTX and HeyA-8/PTX cells, which were induced by miR-137 mimic or si-HULC (Figure 4g and h). These data manifested that HULC knockdown improved PTX sensitivity in SKOV3/PTX and HeyA-8/PTX cells by negatively regulating miR-137.

### 3.5 HULC upregulated ITGB8 expression by negatively interacting with miR-137 in PTX-resistant OC cells

**Figure 5:**
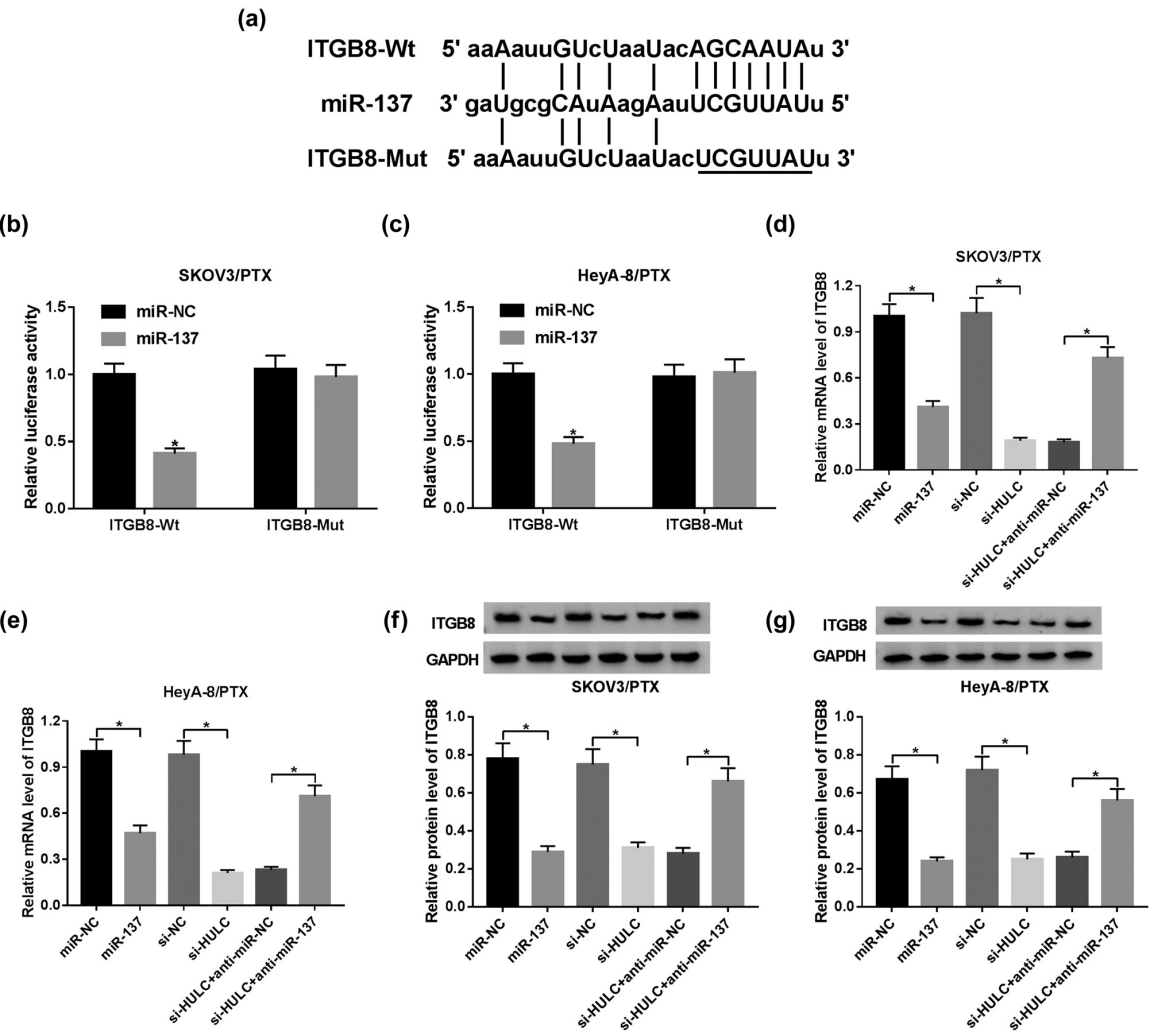
HULC upregulated ITGB8 expression by negatively interacting with miR-137 in PTX-resistant OC cells. (a) The complementary binding sites of miR-137 in ITGB 3′-UTR sequence. (b and c) SKOV3/PTX and HeyA-8/PTX cells were co-transfected with luciferase reporter ITGB-Wt or ITGB-Mut and miR-137 or miR-NC. And the luciferase activities of transfected SKOV3/PTX (b) and HeyA-8/PTX (c) cells were evaluated by dual luciferase reporter assay. (d–g) The SKOV3/PTX and HeyA-8/PTX cells were transfected with miR-NC, miR-137, si-NC, si-HULC, si- HULC + anti-miR-NC, or si-HULC + anti-miR-137, respectively. (d and e) The mRNA level of ITGB8 in transfected SKOV3/PTX (d) and HeyA-8/PTX (e) cells was tested by qRT-PCR. (f and g) The protein level of ITGB8 in transfected SKOV3/PTX (f) and HeyA-8/PTX (g) cells was assessed by western blot. *P < 0.05.

To further explore the potential mechanism of miR-137 in PTX resistance of OC, starBase v2.0 (http://starbase.sysu.edu.cn/starbase2/) was used to search for the putative target of miR-137. The results exhibited that ITGB8 3′-UTR harbored the complementary binding sequences of miR-137 (Figure 5a). ITGB8, a member of the integrin β-chain subfamily, was reported to be highly expressed in several cancers [28,29,30] and was found to be closely related to the chemoresistance in cancer [31]. Subsequently, dual luciferase reporter assay indicated that the luciferase activity of ITGB8-Wt reporter was distinctly reduced in SKOV3/PTX and HeyA-8/PTX cells transfected with miR-137 in contrast with that in the miR-NC group, while the luciferase activity of ITGB8-Mut reporter had no apparent change (Figure 5b and c). Besides, the mRNA and protein levels of ITGB8 were significantly suppressed by transfection of miR-137 mimic or si-HULC, whereas co-transfection of anti-miR-137 and si-HULC relieved the mRNA and protein levels of ITGB8 in SKOV3/PTX and HeyA-8/PTX cells (Figure 5d–g). Taken together, HULC acted as a miR-137 sponge to upregulate ITGB8 expression in PTX-resistant OC cells.

### 3.6 HULC knockdown enhanced PTX sensitivity in PTX-resistant OC cells by decreasing ITGB8 expression

**Figure 6:**
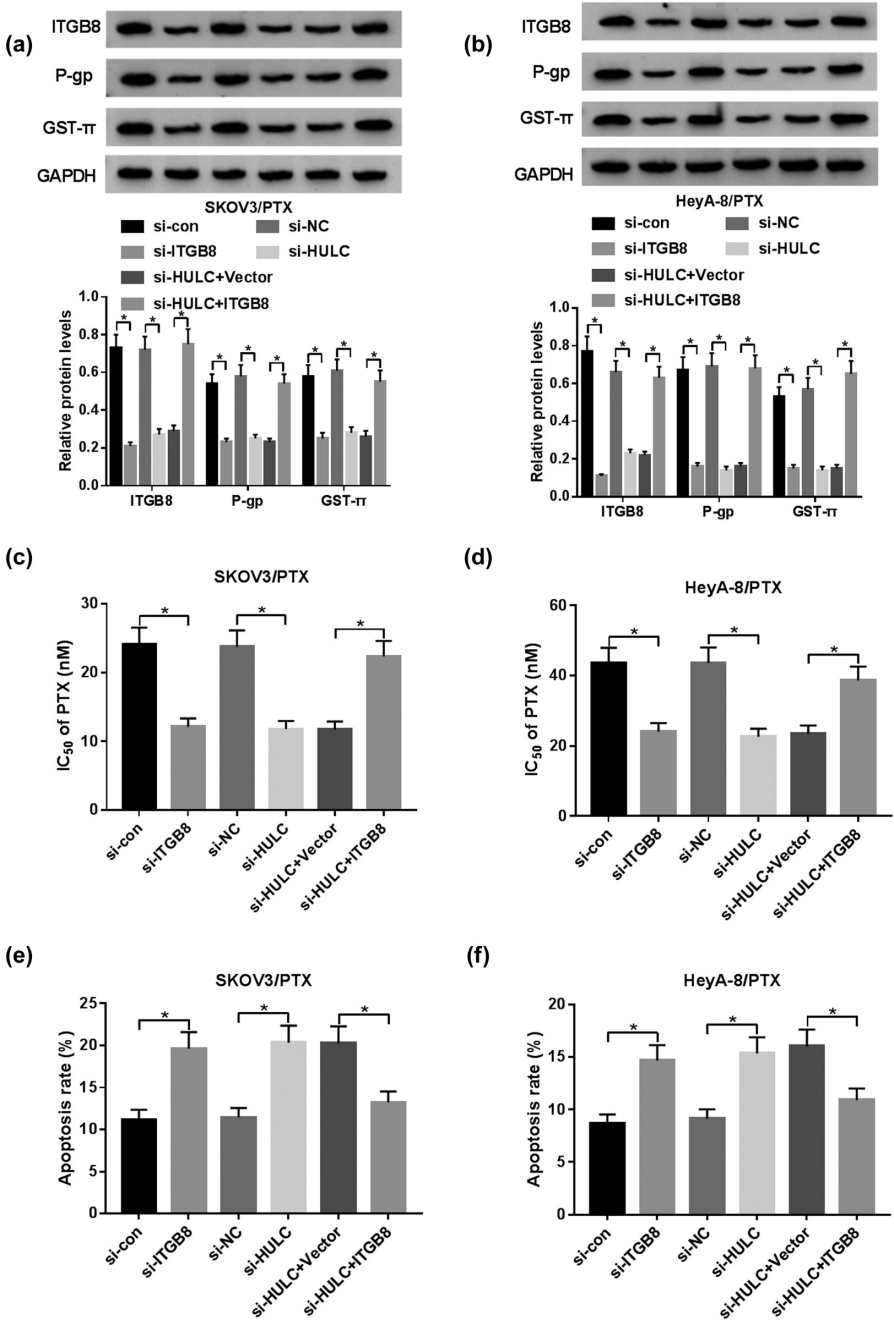
HULC knockdown enhanced PTX sensitivity by decreasing ITGB8 expression in PTX-resistant OC cells. The SKOV3/PTX and HeyA-8/PTX cells were transfected with si-con, si-ITGB8, si-NC, si-HULC, si-HULC + Vector, or si-HULC + ITGB8, respectively. (a and b) The protein levels of ITGB8, P-gp, and GST-π in transfected SKOV3/PTX (a) and HeyA-8/PTX (b) cells were measured by western blot. (c and d) IC_50_ values of transfected SKOV3/PTX (c) and HeyA-8/PTX (d) cells against PTX were assessed via MTT assay. (e and f) The apoptosis rate of transfected SKOV3/PTX (e) and HeyA-8/PTX (f) cells was evaluated through flow cytometry. *P < 0.05.

Next we further explored the relationship between HULC and ITGB8. Western blot assay indicated that the protein levels of ITGB8, P-gp, and GST-π were dramatically reduced by transfection of si-ITGB8 or si-HULC, while this tendency was remarkably alleviated by co-transfection of si-HULC and ITGB8 in SKOV3/PTX and HeyA-8/PTX cells (Figure 6a and b). Besides, IC_50_ values of SKOV3/PTX and HeyA-8/PTX cells against PTX were obviously decreased by transfection of si-ITGB8 or si-HULC, while the transfection of ITGB8 rescued the IC_50_ value of PTX-resistant OC cells against PTX repressed by si-HULC (Figure 6c and d). Furthermore, the apoptotic rate of SKOV3/PTX and HeyA-8/PTX cells was notably elevated by transfection of si-ITGB8 or si-HULC, while it was conspicuously declined in SKOV3/PTX and HeyA-8/PTX cells co-transfected with si-HULC and ITGB8 (Figure 6e and f). These results uncovered that HULC suppressed PTX sensitivity in SKOV3/PTX and HeyA-8/PTX cells via sponging miR-137 to decrease ITGB8 expression.

### 3.7 HULC silencing enhanced PTX sensitivity of PTX-resistant OC *in vivo*

**Figure 7:**
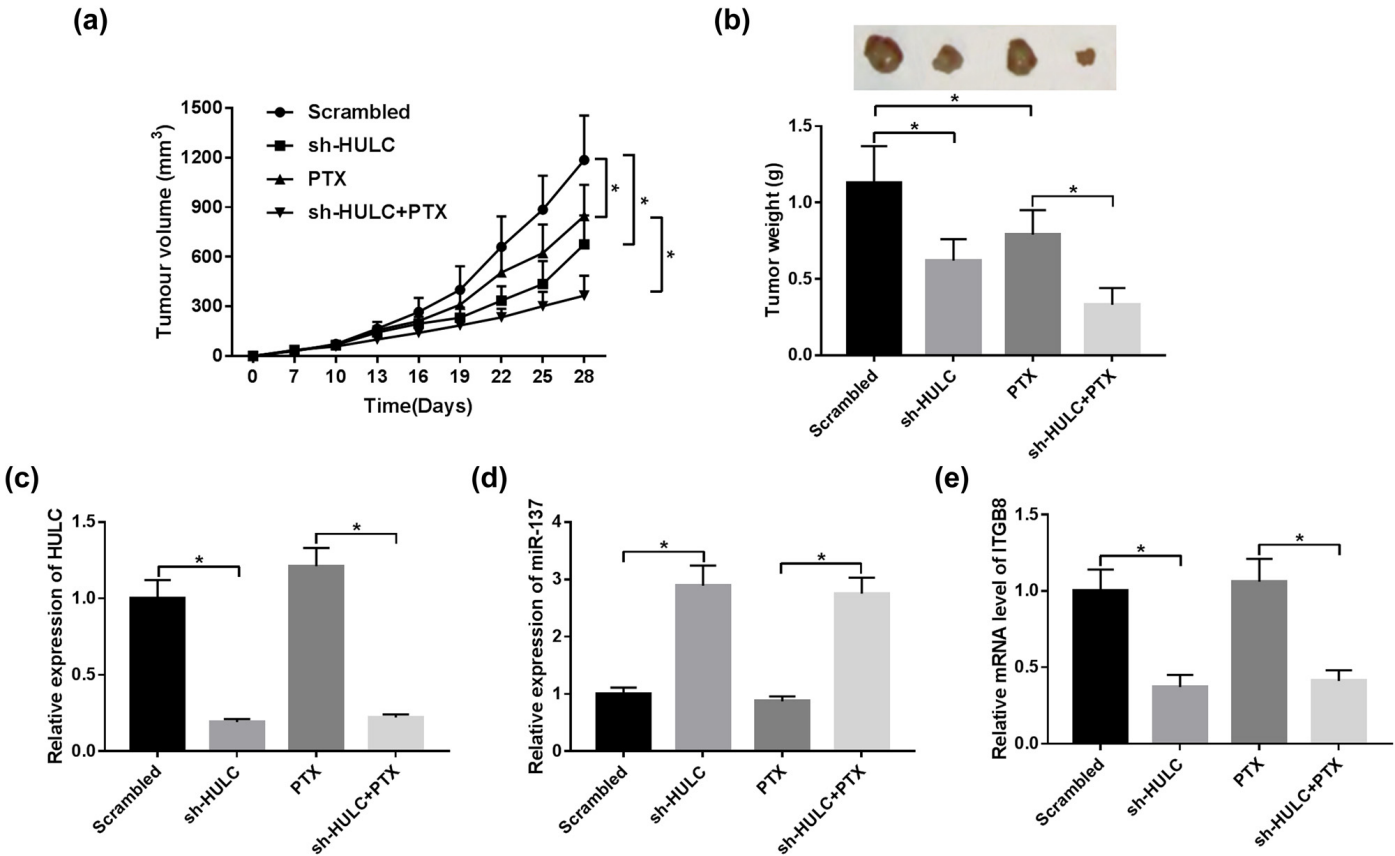
HULC silencing enhanced PTX sensitivity of PTX-resistant OC *in vivo*. Stable SKOV3/PTX cells expressing sh-HULC or control shRNA (Scrambled) were subcutaneously injected into the nude mice (*n* = 6 per group). The mice were injected with PBS or PTX (3 mg/kg) every 3 days for 28 days. (a) Tumor volume of the xenograft tumors in different groups. (b) The weight of xenograft tumors from different groups. (c–e) The expression levels of HULC (c), miR-137 (d), and ITGB8 (e) in xenograft tumor tissues were monitored by qRT-PCR. *P < 0.05.

To further affirm the effects of HULC on PTX resistance *in vivo*, SKOV3/PTX cells stable transfected with sh-HULC or negative control (Scrambled) were subcutaneously injected into the right side of nude mice (*n* = 6 per group). As shown in Figure 7a and b, the volume and weight of xenograft tumor tissues in the HULC knockdown group were distinctly decreased compared with the Scrambled group, and HULC silencing strengthened the suppression effects of PTX on the growth of xenograft tumor (Figure 7a and b). Besides, the expression levels of HULC and ITGB8 were strikingly declined in the sh-HULC group and the sh-HULC + PTX group in contrast with the Scrambled group, whereas the expression level of miR-137 was prominently elevated in the sh-HULC group and the sh-HULC + PTX group (Figure 7c–e). These data suggested that the HULC knockdown improved PTX sensitivity of PTX-resistant OC *in vivo*.

## 4 Discussion

In spite of the effectiveness of PTX in cancer therapy, the acquisition of PTX resistance has been one of the major obstacles in clinical treatment. However, the mechanism underlying PTX resistance remains incompletely understood because of the complex and multifactorial mechanism involved in the chemoresistance regulation. Accumulating evidence has indicated that the aberrant expression of lncRNAs was involved in drug resistance [32,33]. HULC was reported to be associated with the development of chemoresistance in multiple types of cancer. For example, HULC was suppressed by Lentiviral vector carrying METase, and downregulation of HULC suppressed autophagy and enhanced cisplatin sensitivity in drug-resistant gastric cancer cells [34]. Han and Ma indicated that HULC upregulated the expression of myeloid cell leukemia 1 (MCL1) by sponging miR-150-5p, thereby enhancing the resistance of CML cells to imatinib [18]. In the present research, the expression level of HULC was upregulated in OC tissues, and was significantly elevated in PTX-resistant OC tissues and cells. And the expression level of HULC was closely related to the prognosis of OC patients. These research uncovered that HULC might function as a tumor promoter in OC. Besides, PTX-resistant OC cell lines were established to evaluate the effects of HULC on PTX-resistant OC. Rescue experiments disclosed that knockdown of HULC enhanced the sensitivity of PTX-resistant OC cells to PTX treatment via mediating the protein levels of MDR-related proteins and promoting apoptosis *in vitro*. Furthermore, the xenograft model in nude mice exhibited that HULC silencing suppressed the growth of xenograft tumors and enhanced the suppression effect of PTX on xenograft tumor growth *in vivo*. These data implicated that HULC knockdown sensitized OC cells to PTX *in vitro* and *in vivo*.

Further research indicated that miR-137 was a direct target of HULC. Amounting evidence proposed that miR-137 participated in the regulation of chemoresistance in various types of cancer. For example, miR-137 targeted dual specificity phosphatase 4 (DUSP4) in breast cancer to inhibit EMT and suppressed doxorubicin resistance [35]. And miR-137 was suppressed by lncRNA XIST and inhibited 5-FU/cisplatin resistance and glycolysis in colorectal cancer [36]. In accordance with previous research, the expression level of miR-137 was prominently decreased in PTX-resistant OC tissues and cells. Besides, HULC negatively regulated miR-137 level in resistant OC cells, and miR-137 inhibitor partly reversed the effects of HULC on PTX-resistant cells. And we also confirmed that HULC regulated the miR-137 level in OC by acting as a ceRNA for miR-137. Dual luciferase assay indicated the existence of miR-137 response elements in the HULC sequence. And RNA pull-down assay indicated the direct interaction between miR-137 and HULC. Therefore, HULC improved PTX sensitivity of PTX-resistant OC cells by sponging miR-137.

CircRNAs acted as a sponge for miRNA to regulate the downstream mRNA to regulate different signaling pathways. In this research, ITGB8 was a direct target of miR-137, and HULC binds to miR-137 to regulate the expression of ITGB8. ITGB8 is a member of the integrin β-chain subfamily, which was abnormally increased in several types of cancer [28,29,30]. An increasing number of research indicated that ITGB8 was involved in the regulation of chemosensitivity of several human tumors. For example, miR-199a-3p downregulated ITGB8 expression to impede cancer progression, contributing to the improvement of cisplatin sensitivity [37]. Our findings were in-line with the previous research. Overexpression of ITGB8 significantly recuperated the effect of HULC knockdown on the PTX sensitivity of PTX-resistant OC cells. Herein HULC knockdown suppressed the expression of ITGB8 to enhance PTX sensitivity by sponging miR-137 in PTX-resistant OC *in vivo* and *in vitro*.

In conclusion, HULC was highly expressed in PTX-resistant OC tissues and cells, and HULC knockdown enhanced the sensitivity of PTX-resistant OC cells to PTX *in vitro* and *in vivo*. We first identified the HLUC/miR-137/ITGB8 axis in PTX-resistant OC cells, which might shed light on further investigation of the mechanism of chemoresistance.
